# “Pairing assistance”: the effective way to solve the breakdown of health services system caused by COVID-19 pandemic

**DOI:** 10.1186/s12939-020-01190-8

**Published:** 2020-05-15

**Authors:** Tianxiang Chen, Ying Wang, Lei Hua

**Affiliations:** 1 School of Government, Sun Yat-sen University, Guangzhou, Guangdong China; 2 Department of Public Administration, Nanfang College of Sun Yat-sen University, Guangzhou, Guangdong China

**Keywords:** Pairing assistance, Health resources, Health service system, Medical personnel, COVID-19 pandemic

## Abstract

The most terrifying thing about pandemic could be the large number of patients running against the health service system, which causes a serious shortage of health resources, especially medical personnel. Plotting mortality and diagnosis rates against medical staff resources in 16 cities in Hubei Province, where the epidemic was initially concerned and the most severe, shows a significant negative correlation, indicating the critical role of medical staff resources in controlling epidemics. Nevertheless, it is difficult to ensure that there exist enough medical personnel in cities severely hit by the outbreak. China provides solutions by adopting nationwide “pairing assistance” measures with at least one province assisting one city to alleviate pressure in the most severe area. By plotting the number of patients receiving treatment against day, it is clear that implementing “pairing assistance” is a turning point in China’s fight against epidemics.

An epidemic of Coronavirus disease 2019 (COVID-19), which was first identified in Wuhan City, Hubei Province, China in late December 2019, has spread throughout the world rapidly. The coronavirus crisis in China has abated after 2 months of hard work, while the rest of the world has sunk into an increasingly complex quagmire. Scholars own China’s success in controlling COVID-19 to aggressive control measures, such as shutting down industrial and commercial restrictions, monitoring infected person and preventing cross-infection [1–5]. However, the terrifying thing about a pandemic is that the influx of patients overloads the health services system, which leads to a serious shortage of health resources, especially for medical personnel.

By plotting mortality (by Mar 31, 2020) and diagnosis rates (the number of confirmed cases per 1000 population by Mar 31, 2020) against medical personnel resources (the number of medical personnel per 1000 population before the COVID-19 pandemic) in 16 cities in Hubei Province where the virus epicenter in China, we find a significant negative correlation shown in Fig. 1, suggesting the crucial role of medical personnel resources in controlling the pandemic.

**Fig. 1:**
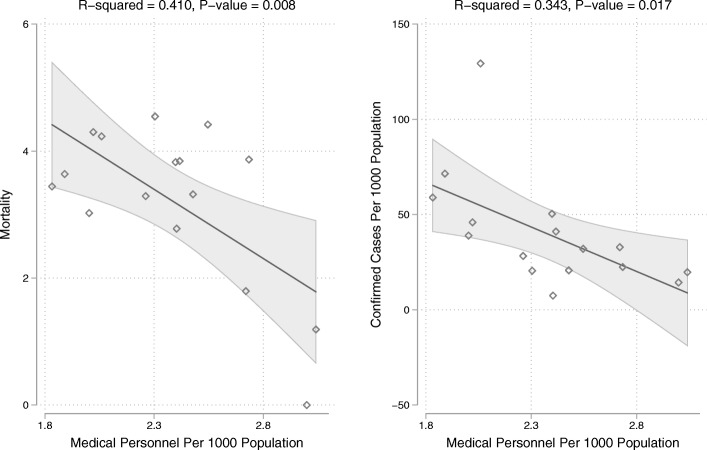
Mortality and diagnosis rates against medical personnel resources

Thus, the medical personnel resources are so important that we should spare no effort to ensure adequate numbers of medical personnel. However, it is difficult for cities severely hit by the outbreak. China offers a solution by taking the nationwide “pairing assistance” measure, mobilizing 29 provinces to alleviate pressure on cities in Hubei province. For example, Wuhan, the provincial capital and the city hardest hit by the disease, had already received 32,572 medical personnel nationwide by Feb 20, 2020. Meanwhile, the other 16 cities with fewer technologies, personnel, and equipment than Wuhan were each partnered with at least one province that has sufficient capacity to assist them (Fig. 2).

**Fig. 2:**
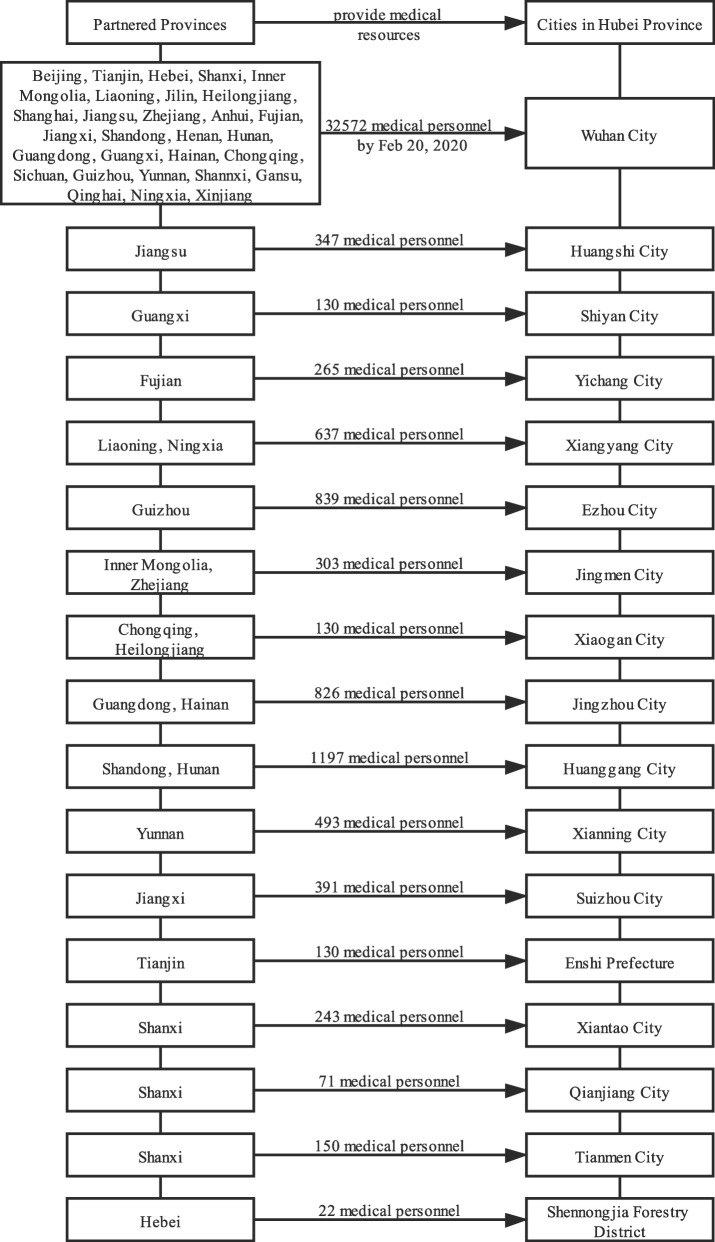
Pairing assistance with 29 provinces mobilized to support cities in Hubei province

The massive mobilization of medical staff, which aims to assist 16 cities besides Wuhan, was started on Feb 11, 2020, while the “pairing assistance” medical personnel got to work in pairing cities from Feb 13, 2020 (the 22nd day after the lockdown of Wuhan). Plotting the number of patients still undergoing treatment against the day clearly showed that the 22nd day after the lockdown of Wuhan was the turning point in fighting the epidemic (Fig. 3) although the total number of confirmed cases was still increasing rapidly at that time, illustrating the evident effect of “pairing assistance” on increasing the cure rate and relieving the pressure on health services system. It is worth noting that Shennongjia Forestry District has a high level of medical personnel resources (2.999 medical personnel per thousand population) and the smallest number of patients (only 11 confirmed cases), so it shows a different curve pattern.

**Fig. 3:**
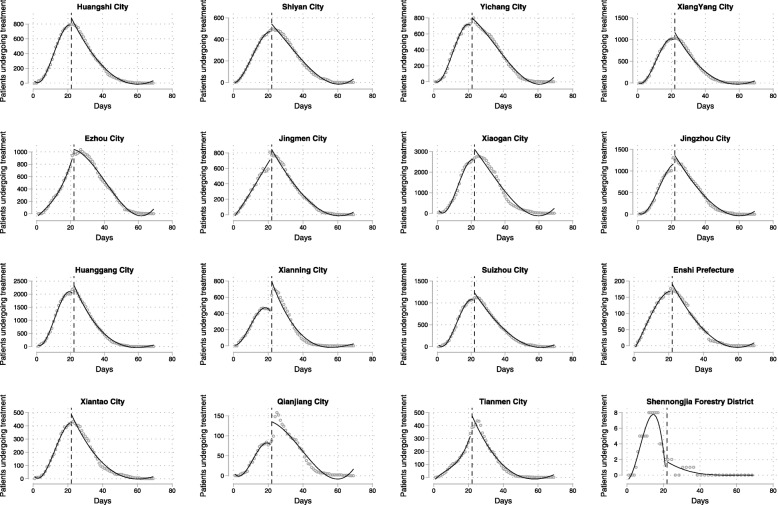
The change of the number of patients still undergoing treatment

In the context of globalization, what we can learn from China’s “pairing assistance” is that the COVID-19 pandemic is not just the business of a single province or a single country. Every sector, every individual and every country must be involved in the fight and support each other. Areas with less severe outbreaks should provide medical resources within their capabilities to the areas with the most severe pandemics. Only by taking the “pairing assistance” measures can we defeat COVID-19, because no one can be immune from the worldwide pandemic.

## Data Availability

The datasets are available from the corresponding author on reasonable request.
